# Impact of Precursor Concentration on Perovskite Crystallization for Efficient Wide-Bandgap Solar Cells

**DOI:** 10.3390/ma15093185

**Published:** 2022-04-28

**Authors:** Shuxian Du, Jing Yang, Shujie Qu, Zhineng Lan, Tiange Sun, Yixin Dong, Ziya Shang, Dongxue Liu, Yingying Yang, Luyao Yan, Xinxin Wang, Hao Huang, Jun Ji, Peng Cui, Yingfeng Li, Meicheng Li

**Affiliations:** 1 State Key Laboratory of Alternate Electrical Power System with Renewable Energy Sources, School of New Energy, North China Electric Power University, Beijing 102206, China; dushuxian10@ncepu.edu.cn (S.D.); 120202211013@ncepu.edu.cn (S.Q.); 120212211057@ncepu.edu.cn (Z.L.); 120212211062@ncepu.edu.cn (Y.Y.); luyaoyan@ncepu.edu.cn (L.Y.); 120202111012@ncepu.edu.cn (X.W.); huanghao@necpu.edu.cn (H.H.); tbyalyf@ncepu.edu.cn (J.J.); cuipeng@ncepu.edu.cn (P.C.); liyingfeng@ncepu.edu.cn (Y.L.); 2 China Three Gorges Corporation, Institute of Science and Technology, Beijing 100038, China; yang_jing12@ctg.com.cn (J.Y.); sun_tiange@ctg.com.cn (T.S.); dong_yixin@ctg.com.cn (Y.D.); shang_ziya@ctg.com.cn (Z.S.); liu_dongxue@ctg.com.cn (D.L.)

**Keywords:** perovskite solar cells, wide-bandgap, precursor concentration, crystallization

## Abstract

High-crystalline-quality wide-bandgap metal halide perovskite materials that achieve superior performance in perovskite solar cells (PSCs) have been widely explored. Precursor concentration plays a crucial role in the wide-bandgap perovskite crystallization process. Herein, we investigated the influence of precursor concentration on the morphology, crystallinity, optical property, and defect density of perovskite materials and the photoelectric performance of solar cells. We found that the precursor concentration was the key factor for accurately controlling the nucleation and crystal growth process, which determines the crystallization of perovskite materials. The precursor concentration based on Cs_0.05_FA_0.8_MA_0.15_Pb(I_0.84_Br_0.16_)_3_ perovskite was controlled from 0.8 M to 2.3 M. The perovskite grains grow larger with the increase in concentration, while the grain boundary and bulk defect decrease. After regulation and optimization, the champion PSC with the 2.0 M precursor concentration exhibits a power conversion efficiency (PCE) of 21.13%. The management of precursor concentration provides an effective way for obtaining high-crystalline-quality wide-bandgap perovskite materials and high-performance PSCs.

## 1. Introduction

Over the last few decades, perovskite solar cells (PSCs) have attracted a lot of research attention due to their ultra-high power conversion efficiency (PCE) and low cost. Currently, the certified PCE of the single-junction PSC reaches 25.7% [1], which is approaching the Schockley-Queisser limit [2]. To further improve the performance of PSCs, tandem solar cells are considered to be an effective path. Generally, wide-bandgap perovskite top cells (1.6–1.9 eV) and narrow-bandgap bottom cells (such as crystalline silicon [3,4], CIGS [5,6], organic solar cells [7] and narrow-bandgap perovskite solar cells [8,9]) are selected to construct efficient tandem solar cells. They are expected to break the theoretical efficiency limit of single-junction solar cells by utilizing photoelectric materials with different bandgaps to realize complementary solar spectral absorption. At present, the technologies of the bottom cell are relatively mature, so it is necessary to conduct in-depth research on the wide-bandgap perovskite top cell.

To further boost the PCE of wide-bandgap PSCs, it is crucial to prepare high-quality wide-bandgap perovskite absorber materials and films. The bandgap of lead-halide perovskite can generally be widened by substituted doping or organic-cation exchange [10,11,12]. For example, Cl or Br elements are used to replace the I element in MAPbI_3_ to adjust the bandgap, which can be continuously tuned from 1.55 eV to 2.3 eV [11,13,14,15]. However, the wide-bandgap PSCs have some challenges, such as the poor crystal quality of perovskite materials, large numbers of trap states in films, and the large open-circuit voltage (*V*_OC_) loss of devices due to the Br-containing compositions, which limit performance [16,17,18,19]. Researchers have made great efforts to improve the perovskite crystal quality, reduce film defects and enhance the PCE by controlling the perovskite crystallization process. For example, the introduction of additives into the precursor [20,21], the regulation of organic cation components [22], and the optimization of film deposition techniques can effectively improve the quality of wide-bandgap perovskite films and reduce the corresponding non-radiative recombination centers [23]. In addition, the concentration of the precursor solution is related to the crystallization, morphology and film thickness of perovskite materials, which can affect the trap states in the film. In fact, the precursor concentration of wide-bandgap perovskite materials is a vital but easy-to-overlook condition for preparing high-performance devices, and the characteristic changes of corresponding device properties are still uncertain. Therefore, studying the influence of the precursor concentration in a simple one-step spin-coating method is of value for further improving the efficiency of the wide-bandgap PSCs. 

In this work, we report the influence of precursor solution concentration on the crystallization characteristics of wide-bandgap Cs_0.05_FA_0.8_MA_0.15_Pb(I_0.84_Br_0.16_)_3_ perovskite film and revealed the corresponding device performance changes. A high concentration of precursor can effectively enhance light absorption and improve the grain size and compactness of perovskite films, but too high a concentration also brings an increase in defects. After balancing the relationship between morphologic structure and defect density, a wide-bandgap PSC with a maximum PCE of 21.13% was achieved at a concentration of 2.0 M. This work reveals the importance of precursor concentration for perovskite crystallization and provides a simple method to improve device performance of wide-bandgap PSCs.

## 2. Materials and Methods

### 2.1. Materials

Formamidinium iodide (FAI), methylammonium bromide (MABr), lead bromide (PbBr_2_), cesium iodide (CsI), 2,2′,7,7′-tetrakis (*N*, *N*-dimethoxy-phenylamino)-9,9′-spirobifluorene (spiro-OMeTAD), 4-tertbutylpyridine (tBP), and lithiumbis-(trifluoromethanesulfonyl) imide (Li-TFSI) were purchased from Xi’an Polymer Light Technology Corp. Lead iodide (PbI_2_, 99.999%) and titanium tetrachloride (TiCl_4_, 99.9%) precursor were purchased from Alfa Aesar. Solvents of dimethyl sulfoxide (DMSO) and *N, N*-dimethylformamide (DMF) were purchased from Acros. Acetonitrile (ACN) and chlorobenzene were purchased from Sigma-Aldrich (St. Louis, MI, USA). FTO glass (12 × 18 mm, 8 Ω·sq^−1^) was purchased from Youxuan New Energy Technology Co. LTD (Yingkou, China).

### 2.2. Fabrication of PSCs

Solution preparation: TiO_2_ precursor was prepared according to a method where 1 mL of TiCl_4_ precursor was diluted with 50 mL deionized water. Then, TiO_2_ precursor was stirred for 1 min. The perovskite precursor of gradients that increased different concentrations of 0.8 –2.3 M were prepared by five solutes of PbI_2_, PbBr_2_, FAI, MABr, CsI in DMF and DMSO (*v*:*v* = 4:1). The prepared precursor solution was stirred at room temperature for 6 h in a N_2_-filled glovebox before use. From 0.8 –2.3 M precursor, the demand for each material was as follows: PbI_2_ was from 307.9 mg·mL^−1^ to 885.3 mg·mL^−1^ with 115.5 mg·mL^−1^ gradient increase, PbBr_2_ was from 48.4 mg·mL^−1^ to 139.2 mg·mL^−1^ with 18.2 mg·mL^−1^ gradient increase, FAI was from 110.0 mg·mL^−1^ to 316.2 mg·mL^−1^ with 41.2 mg·mL^−1^ gradient increase, MABr was from 13.4 mg·mL^−1^ to 38.6 mg·mL^−1^ with 5.0 mg·mL^−1^ gradient increase, and CsI was from 10.4 mg·mL^−1^ to 29.8 mg·mL^−1^ with 3.9 mg·mL^−1^ gradient increase. Specially, excess PbI_2_ of 4 mol% was added to the precursor solution as a defect passivation material. The hole transport layer precursor was prepared by dissolving 80 mg of spiro-OMeTAD powder in 1 mL chlorobenzene with the additives 28.5 μL tBP and 20 μL Li-TFSI (520 mg·mL^−1^ in ACN) [24].

Device fabrication: FTO glass substrates were ultrasonic cleaned with deionized water, ethanol, and acetone for 20 min, respectively. Then, FTO glass substrates were dried by a high-purity nitrogen gas stream. These FTO glass substrates were treated with UV-ozone for 20 min before fabricating the TiO_2_ layer. A compact TiO_2_ film was fabricated using water bath deposition at 70 °C for 60 min according to previous papers [25,26]. After water bathing, the FTO/TiO_2_ film was flushed with deionized water and ethanol and then dried. Then, the FTO glass substrates with compact TiO_2_ film were treated with UV-ozone for 20 min before transferring into the N_2_-filled glovebox (20–27 °C, H_2_O and O_2_ concentrations < 0.1 ppm). Fifty microliters of perovskite precursor solution was dropped on the TiO_2_ film by spinning at 4000 rpm for 40 s. Five hundred microliters of diethyl ether was dropped onto the surface 20 s before the end of procedure. Then, these substrates were transferred onto the heating plate of 100 °C for 15 min in a N_2_-filled inert atmosphere. After the annealing procedure, the substrates were naturally cooled down to room temperature. The size of completed perovskite film was 12 × 18 mm. The spiro-OMeTAD precursor was spin-coated on the perovskite films at a speed of 4000 rpm for 30 s. The Au electrode with a thickness of 60 nm was evaporated on the prepared films in a separate vacuum chamber. Finally, an antireflection coating was deposited on the surface of PSCs.

### 2.3. Characterization of Films and Devices

The current density-voltage (*J*–*V*) characteristics were measured using a source meter (Keithley 2400) under AM 1.5G irradiation with a power density of 100 mW·cm^−2^ from a solar simulator (XES-301S + EL-100) by forward (−0.1 to 1.2 V) or reverse (1.2 to −0.1 V) scans. The light intensity was calibrated with a standard silicon cell (the KG-5 reference cell). All of the devices had no encapsulation and were measured in an ambient environment with 25 °C and ~20% humidity. A mask with an aperture area of 0.071 cm^2^ was applied to test for a more accurate photo-generated current. The external quantum efficiency (EQE) was measured using QE-R systems (Enli Tech. Hsinchu Taiwan China) under AC mode at the same atmosphere as the current–voltage characteristics, and the light intensity was adjusted by the standard single-crystal Si photovoltaic cells before the measurement. The surface morphologies and a section of perovskite films were characterized uniformly by scanning electron microscopy (SEM) (HITACHI SU8100) with a 50.0 k× magnification and a voltage of 1.0 kV and 5.0 kV, respectively. The crystal structure of Cs_0.05_FA_0.8_MA_0.15_Pb(I_0.84_Br_0.16_)_3_ perovskite was analyzed by X-ray diffraction (XRD, Bruker D8 Advance X-ray diffractometer) with a Cu-Kα (λ = 1.54060 Å). The UV-vis spectrophotometer (UV-2600) was used to study the transmittances of precursors and the absorption characteristics of perovskite films in the wavelength of 300 nm to 850 nm consistent with a slit width of 2.0 nm. Photoluminescence (PL) spectra were recorded with a fluorescence spectrometer (FLS 1000) and excitation of 470 nm. All perovskite films with the same preparation process were deposited on the TiO_2_ substrates except the samples prepared for XRD and absorption characteristics, which were deposited on the pure FTO substrates.

## 3. Results

### 3.1. Morphology and Crystallization of the Wide-Bandgap Perovskite Films

**Figure 1:**
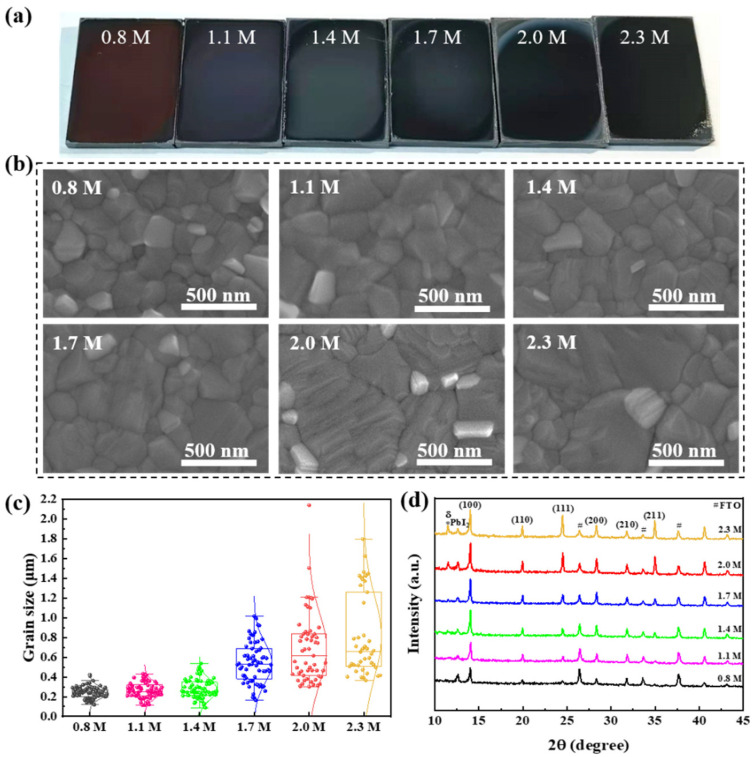
Morphology and crystalline of perovskite films. The perovskite precursor concentrations of perovskite films are 0.8 M, 1.1 M, 1.4 M, 1.7 M, 2.0 M, and 2.3 M, respectively. (**a**) Photographs by camera, (**b**) Top-view SEM images in 50.0 k× magnification, (**c**) Statistical analysis of the grain size in the SEM visual field with same size (2.6 × 1.9 μm), (**d**) XRD patterns (# denotes FTO and * denotes yellow δ phase).

The wide-bandgap perovskite films were fabricated with a mutative precursor concentration. To find out the surface structure of perovskite films, we employed a characterization analysis at different scales. We recorded all of the perovskite films with a camera. From left to right, the concentrations are 0.8 M, 1.1 M, 1.4 M, 1.7 M, 2.0 M, and 2.3 M, as shown in Figure 1a. All the films are relatively flat, whereas the perovskite films of 0.8–1.7 M show a mirror plane, and the films of 2.0–2.3 M show a slightly frosted surface. From the concentration of 0.8 M to 2.0 M, the color of wide-bandgap perovskite films gradually deepens, and the 2.3 M is the same as 2.0 M. It is speculated that the color transformation of perovskite films may be connected with the thickness or surface structure of perovskite films, which are likely affected by the variation of precursor concentration. To confirm this speculation, we performed a microscopic characterization by SEM.

To observe the crystallization of thin film distinctly, directly, and accurately, the perovskite films with various precursor concentrations were characterized by SEM, as shown in Figure 1b. In these samples, the perovskite composition was Cs_0.05_FA_0.8_MA_0.15_Pb(I_0.84_Br_0.16_)_3_ and all films were annealed before SEM testing under the same temperature. SEM images with a 50.0 k× magnification show that perovskite films with all precursor concentrations have dense and whole grains. The compact morphology of perovskite films can effectively avoid the generation of leakage current of PSCs. The grain size increases with increasing precursor concentration, and the number of disorderly grains decreases. A statistical analysis of the grain size in the SEM is shown in Figure 1c. We counted the grain sizes in the visual field that were the same size (2.6 × 1.9 μm). The grain sizes of 0.8 M, 1.1 M and 1.4 M samples are similar (most grain sizes distribute in 0.2–0.35 μm), while the grain size of 1.4 M appears 0.5 μm. The average grain size of 1.7 M is more than 0.5 μm, and the maximum value is 1 μm. The films of 2.0 M and 2.3 M samples have numerous large grains with the grain size above 1.2 and 1.4 μm, respectively. This demonstrates that a higher precursor concentration is beneficial to grain growth. As the precursor concentration increases, the supersaturation increases, which would result in larger nuclei. With the annealing process, the grains begin to grow and become larger due to the higher supersaturation [27]. Then, according to the Ostwald ripening process, the high solubility and surface energy of the small-sized particles facilitate their dissolution and deposition on larger particles, while the larger particles grow, and the average particles increase [28,29,30,31,32]. This process explains the phenomenon of the grain size increasing with the increase in precursor concentration. The grain growth, along with the reduction in grain boundaries, is also beneficial for reducing trap-induced charge loss at the grain boundaries [33,34]. The perovskite films with a 0.8 M concentration possess a small grain size (below 350 nm) and clear grain boundary. As the perovskite precursor increases to 2.0 M, more texture resulting from the crystal growth process can be observed at the surface of grains. The above phenomenon stresses that the variations of perovskite precursor concentration affect the crystallization and morphology of films because these films were fabricated under the same conditions, except for precursor concentrations. In addition, XRD patterns also confirm the impact of perovskite precursor concentration on the crystallization of perovskite films.

The crystalline quality of perovskite films is characterized by XRD. The perovskite films are deposited on the FTO substrate, which can analyze the chemical compositions and crystal structures of perovskite films. As shown in Figure 1d, a series of diffraction peaks are located at 2θ = 14.0°, 19.9°, 24.5°, 28.3°, 31.7°, 34.9°, and the crystal faces corresponding to peak positions are marked. All perovskite films show similar peak positions, which are consistent with typical perovskite films [35,36]. It can be shown that the change in concentration does not change the material composition of the wide-bandgap perovskite film. The peak intensity indicates the great crystallinity of perovskite films. The peaks of PbI_2_ appear at all the concentration gradients due to additional PbI_2_ of 4 mol% in the precursor solution. Numerous reports indicate that appropriate excessive PbI_2_ can effectively reduce the defect density of perovskite films and inhibit the recombination of carriers [37]. When the concentration exceeds 1.4 M to 2.3 M, a perovskite peak (2θ = 11.5°) is observed and more obvious in the sample of 2.0 M and 2.3 M. According to various reports, the hexagonal delta phase (yellow δ phase) can be observed at 2θ = 11.7° in the perovskite dominated by FAPbI_3_. Our Cs_0.05_FA_0.8_MA_0.15_Pb(I_0.84_Br_0.16_)_3_ perovskite was also dominated by FAPbI_3_ [38,39]. Therefore, we speculate that the peak of 2θ = 11.5° is likely a hexagonal delta phase. Although the appearance of the yellow δ phase perovskite may result in poor phase stability, some studies have shown that the favorable energy level arrangement of the α/δ junction significantly inhibits the charge recombination [40,41]. Briefly, the changes in concentration do not clearly affect the growth orientation of the perovskite film.

### 3.2. Optical Properties of the Wide-Bandgap Perovskite Films 

**Figure 2:**
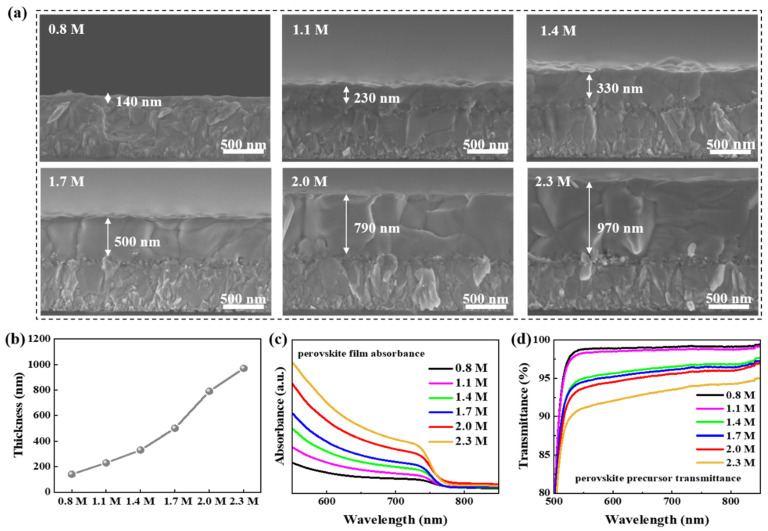
Absorption characteristics of perovskite films with different perovskite precursor concentration, 0.8 M, 1.1 M, 1.4 M, 1.7 M, 2.0 M, 2.3 M. (**a**) Cross-section SEM images, (**b**) Thickness of perovskite films, (**c**) Absorption spectra of perovskite films, (**d**) Transmittance spectra of perovskite solution.

The absorption of perovskite film depends not only on the crystallization of the film but also on the thickness of the film. To further reveal the crystalline property inside the perovskite film and ensure film thickness, we carried out cross-section SEM measurements on the perovskite film with different precursor concentrations. As shown in Figure 2a, all the films exhibit a density cross-section morphology. After analyzing them in detail, perovskite films of all concentrations exhibited crystallization characteristics consistent with those described above. A perovskite film fabricated with 2.0 M precursor possesses a better crystalline property with a penetrative crystal vertically to the substrate. The grain quality of perovskite film fabricated with 2.3 M precursor is inferior to 2.0 M due to the incoherent grains and cavities at the interface of FTO substrates and perovskite layers. Hence, the precursor concentration of 2.0 M is more likely to possess high-quality perovskite film. 

As shown in the cross-section images of the perovskite films, we further characterized the thickness of the films and marked them with white words. To visualize the film thickness changing with the different precursors, we systematically summarized the thickness data and plotted a thickness–concentration diagram, which is shown in Figure 2b. It is obvious that the thickness shares a similar tendency with the precursor concentration, and the film thickness increases with the increase in precursor concentration. In the case of thickness increasing with concentration, the perovskite film of 2.3 M has the maximum thickness. Even so, it is worth noting that the precursor with a higher concentration than 2.3 M, such as 2.6 M, appears colloidal and has a greater viscosity, making it difficult to filter, drip and spin. It is speculated that film thickness is related to absorption characteristics. To further explore the influence of precursor concentration on perovskite film absorption, we investigated the optical property of films and perovskite precursors by UV-vis-NIR spectroscopy measurements. The absorption spectra of wide-bandgap perovskite films from 550 nm to 850 nm are shown in Figure 2c. The light absorption of perovskite film is positively correlated with film thickness. The perovskite film of 2.3 M exhibits the highest absorption, which confirms the inference that film thickness has a positive effect on film absorption. As shown in Figure 2d, the transmittance of the precursor solution with different concentrations is also measured; in the visible region, all precursors have a high transmittance, which is much higher than that at the near-ultraviolet region. In brief, as the concentration increases, the transmittance of the precursor decreases, and the absorption of the perovskite film increases.

Combining the film thickness data, a view is put forward that the precursor with a high concentration can fabricate a perovskite film with a high thickness, and a thicker film is prone to obtain a high absorption. High absorption is critical for efficient PSCs, so it is expected that proper precursor concentration regulation is conducive to the improvement of PCE. Although the thickness and absorption of the perovskite films increased with the increasing precursor concentration, the bandgap of perovskite materials always remained around 1.6 eV. In summary, this section stresses the importance of precursor concentration for the absorption and crystallization of perovskite films.

### 3.3. Trap States of the Wide-Bandgap Perovskite Films 

**Figure 3:**
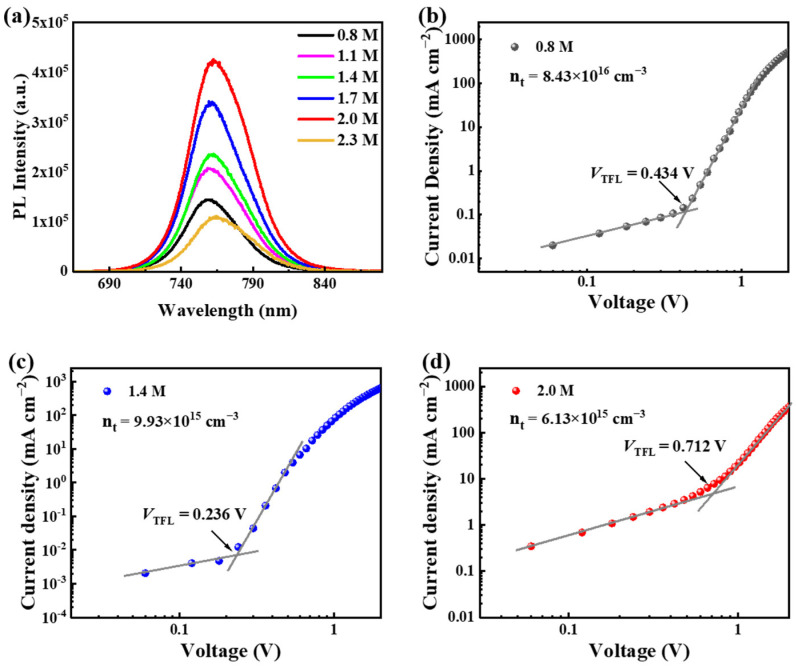
Defect density analysis. (**a**) Steady-state PL spectra of glass/FTO/perovskite. (**b**–**d**) The space-charge-limited current curves of the electron-only devices with a structure of FTO/TiO_2_/perovskite/PCBM/Au.

The defects of perovskite films are evaluated by steady-state PL spectra from 0.8 M to 2.3 M, based on the structure of glass/FTO/perovskite and the space-charge-limited current curves of the electron-only devices with a structure of FTO/TiO_2_/perovskite/PCBM/Au. The PL intensity reflects the intensity of electron and hole compound luminescence in a quasi-equilibrium state after light excitation. That is to say, the stronger the PL intensity, the easier it is to compound luminescence, which means much lower trap states. According to Figure 3a, all the samples exhibit an excellent photoelectric property, indicating a good crystalline quality of perovskite films with different concentrations, which is in agreement with the results of SEM and XRD. As the concentration of the precursor increases, PL intensity shows a trend of first increasing from 0.8 M to 2.0 M in sequence but then decreasing rapidly at 2.3 M. The difference between PL spectra mainly results from the defects (such as I^−^ vacancy, MA^+^ vacancy, interstitial I^−^) that are hard to characterize using SEM and XRD measurements. So, even though the perovskite films exhibit similar grains and XRD patterns, their intensity of PL spectra is different. PL intensity shows that the perovskite films with 2.0 M concentration exhibit the greatest radiative capacity, implying that both the non-radiative composite inside the film and defects are the lowest. This result is consistent with the SEM images analysis above. The enlarged whole grains and reduced grain boundaries facilitate the quality of perovskite films. Once again, the impact of precursor concentration on the crystallization of perovskite film is demonstrated. 

To comprehensively explore the effect of precursor concentration on perovskite film quality, we characterized the intrinsic defects of the films, which formed during the homogenization process. As shown in Figure 3b–d, to further prove that the defect concentrations gradually decrease between 0.8 M and 2.0 M, we obtained the dark-state *J*–*V* test of the single-electron transport layer of the perovskite solar cell prepared by the precursors with concentrations of 0.8 M, 1.4 M, and 2.0 M, respectively. PCBM and a compact TiO_2_ layer play a role in the upper and lower electron transport layers, respectively. The defect density values of electronic defects in perovskite films prepared with different precursor concentrations do not show a large difference, while those of defect concentrations can be significantly decreased from 8.43 × 10^16^ cm^−3^ to 6.13 × 10^15^ cm^−3^, further supporting the above discussion that the perovskite films prepared with 2.0 M precursors have fewer defects.

### 3.4. Performance of Wide-Bandgap PSCs

**Figure 4:**
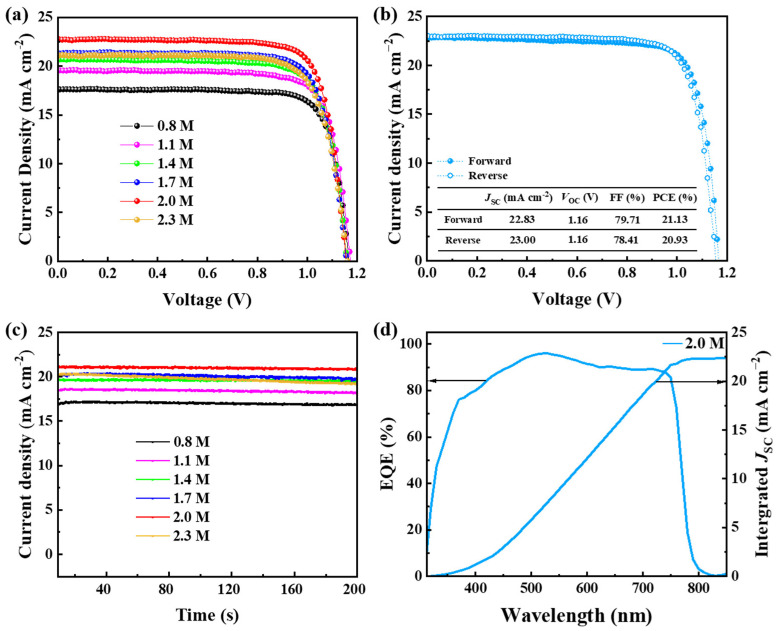
Performance characteristics of PSCs. (**a**) Current–voltage characteristic curves of devices with different precursor concentrations. (**b**) Current–voltage characteristic curves (including forward and reverse scan) of champion PSC. (**c**) Maximum power point (MPP) tracking of PSCs. (**d**) EQE spectra and corresponding integrated *J*_SC_ of the device with 2.0 M precursor concentrations.

After finding out the influence of the wide-bandgap perovskite precursor concentration of the crystallization of films, we investigated the effect of the precursor solution concentration on photo-voltaic performance by fabricating and testing PSCs with different concentrations of precursor solution. The structure of PSCs is FTO/TiO_2_/perovskite/spiro-OMeTAD/Au, and the wide-bandgap perovskite active layer was prepared by one-step spinning with diethyl ether as anti-solvent. Transparent conductive substrates of FTO and Au are selected as the electrode. The contact TiO_2_ layer is used as electron transfer layer, and spiro-OMeTAD serves as the hole transfer material. The impact of the precursor concentration on the performance of perovskite solar cells is primarily shown by the *J*–*V* curves of the PSCs as shown in Figure 4a. The current density (*J*_SC_) of others concentrations substantially increases compared with the 0.8 M precursor solution. The champion PCE of 21.13% is simultaneously achieved on 2.0 M precursor concentration and the *J*_SC_ of 22.85 mA cm^−2^. As shown in Figure 4b, the subtle difference between forward and reverse scans of champion PSC indicates that hysteresis is noteless. The maximum power point (MPP) tracking is shown in Figure 4c. Devices at all precursor concentrations exhibit a high stabilized current density, which reflects good operation stability. The variation of precursor concentration causes no significant changes in device power output. As shown in Figure 4d, the corresponding EQE of ≥90% was achieved over a wide wavelength range from 460 nm to 720 nm, which gave an integrated *J*_SC_ of 22.57 mA cm^−2^ that was consistent with the *J*–*V* values. The integrated *J*_SC_ is comparable to that of the *J*_SC_ determined from the *J*–*V* curves. Table 1 shows the photovoltaic performance of PSCs fabricated at different precursor concentrations. The champion performance of PSCs was acquired by the synthetic action of optimal precursor concentration of 2.0 M on high-quality, wide-bandgap perovskite films. 

**Figure 5:**
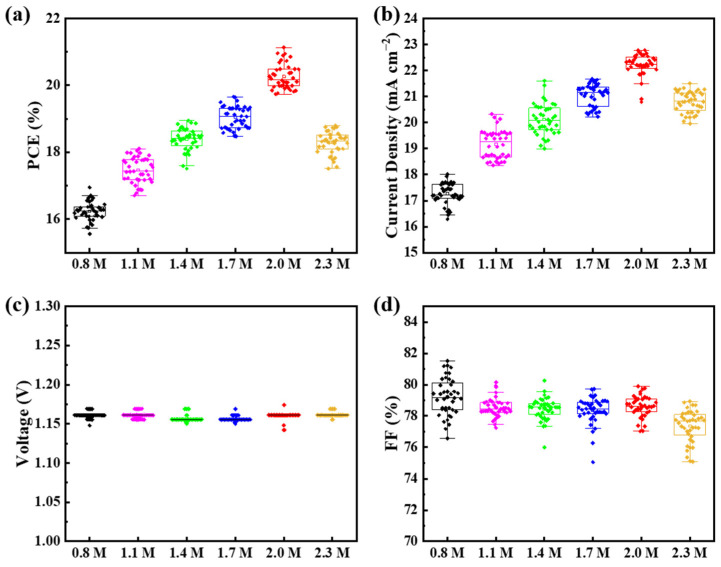
Performance distribution of PSCs. (**a**) PCE, (**b**) *J*_SC_, (**c**) *V*_OC_, (**d**) FF.

The distribution of PCE, *J*_SC_, *V*_OC_, FF is intuitively shown in Figure 5, which are selected from eight groups of the same experiments. The obvious variation parameters are *J*_SC_ and PCE. The change in *J*_SC_ is ascribed to the increase in crystallization quality and film thickness, and is consistent with the analysis of perovskite film properties above.

## 4. Conclusions

In conclusion, an essential but easily overlooked factor of the precursor concentration of wide-bandgap perovskite crystallization was explored. The precursor concentration affects the perovskite film quality by influencing morphology, crystallinity, optical property and defect density. A high precursor concentration causes the following processes—the increase the grain size, improvement in the crystalline quality and absorption of perovskite films—to decrease the defects of perovskite films, which enhance the performance of perovskite films. As a result, the champion PCE of 21.13% is achieved in the PSCs with a 2.0 M precursor concentration based on Cs_0.05_FA_0.8_MA_0.15_ Pb (I_0.84_Br_0.16_)_3_ wide-bandgap perovskite material, which is attributed to high crystalline quality and fewer defects. Meanwhile, the interrelated PSCs show excellent reproducibility. The high-quality perovskite films with large grains and a high crystallinity can reduce the defects and improve the mobility of carriers. This work provides an impressive reference for improving the crystallization of wide-bandgap films and elevating the performance of PSCs.

## Figures and Tables

**Table 1:** Photovoltaic performance of PSCs fabricated at various precursor concentrations.

Precursor Concentration	*J*_SC_ (mA cm^−2^)	*V*_OC_ (V)	FF (%)	PCE_best_ (%)	PCE_average_ (%)
0.8 M	17.62	1.16	79.53	16.38	16.08 ± 0.35
1.1 M	19.56	1.16	78.92	18.05	17.45 ± 0.67
1.4 M	20.70	1.15	78.11	18.69	18.35 ± 0.59
1.7 M	21.31	1.15	78.45	19.31	18.92 ± 0.46
2.0 M	22.85	1.16	79.71	21.13	20.39 ± 0.74
2.3 M	21.18	1.16	76.18	18.73	18.42 ± 0.42

## Data Availability

All data will be available on request from the corresponding authors.
